# Treatment of Children’s Mouths

**Published:** 1907-05

**Authors:** Geo. S. Tignor

**Affiliations:** Atlanta, Ga.


### TREATMENT OF CHILDREN'S MOUTHS.

GEO. S. TIGNER, A.B., D.D.S., Atlanta, Ga.

---

Given a good set of teeth, well-placed and well-kept, almost any face is presentable, most faces are attractive, and many are beautiful; on the other hand, unclean mouths, malposed teeth, vulgar displays of gold, or missing teeth, disfigure any face.

How often have you been disenchanted after having been charmed by a face that was otherwise attractive, when, at her first word, she flashed a gold crown or displayed a mouth full of neglected, discolored teeth? Such mouths are objectionable to most people and positively repulsive to the refined.

When it is in our power to prevent or correct these deformities, it is our duty to society to do so.

Prophylactic treatment of children's mouths will best conserve this end. Massaging the gums with antiseptic and stimulating mouth washes, will aid babies in their first dentition.

This simple treatment maintains normal circulation through the soft tissues of the mouth, thus preventing local inflammation, attended with fever, fretfulness, refusal of nourishment, diarrhœa and convulsions.

This treatment will possibly prevent the various forms of stomatitis at different ages of children.

It is a Japanese aphorism that if both ends of the alimentary track are kept in health, the intervening section will take care of itself. It seems to me that the health of the entrance to this important canal is of the greatest interest to us all. A stream will never be clear as long as you disturb the fount. Let's help each other keep the children healthy. Decay of teeth doesn't occur in clean mouths.

The dentist wants the co-operation of the family physician in seeing that the mouths of the children of their clientele are brought to the dentist for examination early and often, in order to prevent this dreaded decay.

The application of prophylaxis is of most value during the period when children's deciduous teeth begin to decay and the first permanent molars come. The ignorance of parents about the erupton of the sixth-year molars is the cause of their loss more than that of any other teeth in the mouth; and their loss is more deplorable because they are the best grinders we ever have, and give more character to the general contour of our faces than any other teeth. At this age, the health of more children is lost from neglect of their mouths than is hurt by our impure milk and food supply, or is damaged by our unsanitarw school houses.

It is important that we guard the cleanliness of our milk supply and see that our school-rooms are filled with fresh air, but let's not forget the pearly gateway to the young and delicate constitutions of our children.

Many children are admitted to our public schools whose mouths carry 1,140,000,000 micro-organisms. I make this statement upon the authority of Dr. W. D. Miller, of Berlin, whose ability as a bacteriologist is well known. If tubercle bacilli are so often found in our milk supply, isn't it plausible that they may be found in large numbers in the neglected mouths of our children?

Our progressive physicians and enterprising newspapers are making a noble fight for sanitary school houses, which is timely and should be effective.

If there are many evidences that the coming summer is to bring conditions which make the care of public health one of the highest and most responsible duties of the city's officials, what shall we do about these unclean mouths?

Wisdom and prudence would suggest that we guard the purity of our children's food unceasingly and that we purge our school buildings of foul air and insanitary surroundings before the heat of the summer sun quickens to life germs which may be militant and destructive, even to epidemics before mid-summer days.

The mouths of these innocents carrying carious deciduous teeth, calcarious deposits, suppuration, lacerated and infected soft tissues supplied with abundant warm moisture, furnish ideal hotbeds for germinating an endless variety of bacteria. Can you imagine more fertile ground for their multiplication and development?

Food, however pure, when moistened and ground up in such a charnal house, will carry more bacteria to the stomach and intestines than they can resist; or the child, in order to avoid pain, will bolt his food habitually, thus overtaxing other organs of digestion.

Cases are on record where the unobservant stomach specialists have wrought in vain against the poisoning effects of a flow from alveolar abscesses in the mouths of their patients.

Cases are on record where pathological conditions in the mouth resulting from local causes have confused diagnosis and brought two-fold sorrows.

Any lowered vital or abnormal condition of the body is manifested in the mouth, and there is scarcely any pathological condition that an inflamed or suppurating condition of the mouth will not accelerate or produse.

If this be true, the dentist and physician have much need of each other's help daily. The study of the relation of oral sepsis and general diseases is yet in its infancy, but the fact that increased knowledge of pathology, and especially of bacteriology, is leading the medical profession to place an increasing significance upon the condition of the mouth as a factor both in the production and prevention if diseases, justifies us in the hope that oral prophylaxis may soon be regarded as so important that our public schools, hospitals and institutions for the treatment of pulmonary tuberculosis, may avail themselves of the help of the dental surgeon.

We do not claim that operative work in restoring lost parts in the mouth will endure or prevent systemic diseases, without oral asepsis. Surgical cleanliness must be maintained in the mouth, and the family physician occupies the relation that puts the burden of responsibility on his shoulders.

Every day the progressive dentists are realizing that the observance of aseptic surgical methods are imperative. If by taking thought, we can make the race uniformly better looking, help the children through a tender age, simplify the diagnosis of so many diseases, and improve the conditions for the health and happiness of the next generation, let us think on these things.

